# PMTNet: A Part-Centric Missing-Aware Temporal Network for Cat Behavior Recognition in Unconstrained Videos

**DOI:** 10.3390/ani16111589

**Published:** 2026-05-23

**Authors:** Chunxi Tu, Jiatao Wu, Zeguang Huang, Jiaxing Xie

**Affiliations:** 1 College of Artificial Intelligence and Low-Altitude Technology, South China Agricultural University, Guangzhou 510642, China; 202228110721@stu.scau.edu.cn (C.T.); wjt_@stu.scau.edu.cn (J.W.); 2 College of Water Conservancy and Civil Engineering, South China Agricultural University, Guangzhou 510642, China; zeguang@stu.scau.edu.cn; 3 Engineering Research Center for Monitoring Agricultural Information of Guangdong Province, Guangzhou 510642, China

**Keywords:** cat behavior recognition, animal behavior analysis, DEIM-based detection, part-centric temporal modeling, missing-aware fusion

## Abstract

Cat behavior recognition in unconstrained videos is important for animal welfare monitoring and veterinary assessment, yet remains challenging because behavior cues are often carried by highly deformable and intermittently visible parts such as the head and tail. This study aims to improve clip-level cat behavior recognition under unstable part visibility in real-world videos. We propose PMTNet, a part-centric temporal network for cat behavior recognition under unstable part visibility. The framework first detects the cat body, head, and tail using a DEIM-based detector, then selects a detector according to video-domain continuity and stability, and finally models behavior from ROI appearance features and explicit geometric motion cues. The framework was developed and evaluated using a part-detection dataset of 4000 training images and 500 validation images, together with a cat behavior dataset of 1283 video clips across five categories. In the best-performing setting, PMTNet achieved 93.1% Top-1 Accuracy and 90.9% Macro-F1. Ablation studies further suggest that detector choice in the video domain, complementary part cues, and missing-aware fusion all contribute to the final recognition performance. On the present dataset, PMTNet also outperformed representative end-to-end video recognition baselines. These results support the use of part-centric temporal modeling for cat behavior recognition in unconstrained real-world videos.

## Simple Summary

Cats show changes in health, stress, and welfare through everyday behavior, but recognizing these behaviors from ordinary video is not easy. In real-world recordings, cats bend and twist, and important clues from the head and tail may be blurred, partly hidden, or briefly lost. In this study, we aimed to improve video-based cat behavior recognition by looking at the body, head, and tail separately and then combining this information over time. We prepared 4500 labeled images for finding the cat, head, and tail, and a dataset of 1283 video clips covering five common behavior groups. In our experiments, the proposed method reached 93.1% overall accuracy and performed better than several commonly used video analysis approaches. These findings suggest that paying attention to different body parts can make cat behavior recognition more reliable in everyday conditions. This may support future tools for animal welfare monitoring, veterinary observation, and long-term care in homes, clinics, and shelters.

## 1. Introduction

Cats often express changes in health, stress, and welfare through everyday behavior, making reliable behavior recognition valuable for feline welfare assessment, veterinary observation, and long-term monitoring [1]. With the growing availability of real-world video, vision-based analysis has become an attractive non-invasive direction for animal behavior understanding. However, methods developed primarily for human action recognition do not transfer well to cats [2].

Cats can bend and stretch their flexible backs during movement, giving their motion a “liquid-like” appearance [3]. As a result, cat behavior is often expressed not only through global body movement but also through subtle motion of specific anatomical parts. In particular, head and tail cues are often behaviorally informative, but they are difficult to detect reliably in ordinary videos [4,5,6]. The tail, in particular, is small, moves quickly, and is often blurred or occluded [7,8]. When these parts are missed in some frames, the temporal evidence needed for clip-level recognition becomes incomplete.

Existing video recognition methods usually process the whole frame or the whole video clip [9,10,11]. These models work well in many human action-recognition benchmarks, but they can be less reliable in real-world animal videos [2,12]. One reason is that full-frame models may learn from background or scene context instead of the animal’s actual movement [13,14].

Skeleton-based methods provide another option because they focus on body structure rather than the whole image. However, stable animal pose estimation is still difficult in videos with occlusion, interaction, and large body deformation [8,15,16,17]. For cats, dense fur, self-occlusion, and flexible body shapes make keypoint extraction especially challenging. Therefore, the main problem is how to recognize behavior from local anatomical cues when these cues are not always visible or stable over time.

A practical compromise is to use bounding boxes for important body parts, such as the body, head, and tail. This representation helps reduce background interference [18,19] and avoids the need for dense keypoint annotation, which remains challenging for animal pose estimation and tracking [7]. It also allows the model to focus on behavior-related local regions without requiring precise joint localization [20]. However, this part-based representation also brings a new challenge: the detected boxes must remain reliable over time. In real-world videos of cats, body deformation, intermittent tail missingness, and bounding-box jitter can make part trajectories unstable. Such instability may reduce the reliability of downstream temporal behavior recognition [21].

Within feline research, automated behavior analysis remains limited [22,23]. Early veterinary and ecological efforts primarily used wearable inertial sensors to classify basic locomotor states [22]. These devices provide continuous data but may also cause stress and alter the animal’s natural movement patterns. Recent non-invasive visual approaches have largely focused on static image analysis, such as feline pain assessment through facial grimace scales [24,25]. However, static imagery cannot capture the temporal dynamics of complex activities such as grooming or active play [2]. Existing video-based feline monitoring systems typically treat the cat as a single bounding box for indoor location tracking. Although this captures global spatial trajectories, it provides limited local anatomical information, such as head or tail cues, for distinguishing specific behaviors [26,27]. A dedicated visual framework for parsing the non-rigid, multi-part temporal dynamics of cats in unconstrained videos remains underexplored.

To address this challenge, we propose PMTNet for clip-level cat behavior recognition in unconstrained videos. The key idea is to treat the cat body, head, and tail as separate but complementary sources of evidence. PMTNet does not assume that every part can be detected reliably in every frame. Instead, it explicitly models missing or unstable part observations during temporal fusion. This design allows the model to use local behavioral cues while reducing dependence on background information or dense keypoints. The main contributions of this work are as follows:

(1) We present PMTNet, a part-centric missing-aware temporal framework for cat behavior recognition in unconstrained videos. PMTNet uses three part streams: the cat body, head, and tail. These streams provide complementary evidence for behavior recognition. The model combines region-of-interest (ROI) appearance features with explicit geometric motion descriptors, instead of relying only on full-frame video, a single cat box, or dense skeleton keypoints.
(2) We introduce a downstream-aware detector selection protocol for temporally structured animal-video tasks. Instead of selecting part detectors solely by static image-domain metrics such as mean average precision (mAP), We evaluated candidate detectors using video-domain missingness, continuity, and spatial stability of part trajectories. The final detector was also tested in the downstream behavior-recognition task. In this way, detector selection was linked to final temporal recognition performance, rather than only to frame-level detection accuracy.
(3) We establish a real-world experimental benchmark for this task, consisting of 4500 annotated images for part-level detection and 1283 labeled video clips across five behavior categories, and provide systematic ablations and baseline comparisons that clarify the contributions of detector robustness, complementary part modeling, missing-aware fusion, and temporal aggregation.

## 2. Materials and Methods

### 2.1. Dataset and Data Collection

This study used two types of data: (i) image-based annotations for part-level detection and (ii) short video clips for downstream behavior recognition. The image dataset is used to train the part detector, whereas the video dataset is used to evaluate and train the temporal behavior recognizer.

#### 2.1.1. Image Dataset for Part-Level Detection

We constructed a Common Objects in Context (COCO)-format detection dataset with three categories: cat, head, and tail. The dataset contained 4000 training images and 500 validation images. Images were collected from two sources: (1) original images captured by the authors in natural daily environments, and (2) publicly available resources released under permissive licenses.

To avoid potential overlap between the part-detection image dataset and the behavior video dataset, we conducted source-level and visual de-duplication checks during data curation. For self-collected materials, images and videos were organized according to recording source, acquisition session, and scene context. For publicly available materials, identifiable source records and visual content were also checked. Exact duplicates and visually near-duplicate samples across the detection-image and behavior-video datasets were screened during this process. After this de-duplication procedure, no overlap was found between the part-detection image dataset and the behavior video dataset.

Because the dataset combines self-captured and publicly sourced imagery, the acquisition devices are heterogeneous, and device metadata were not consistently retained in the released COCO annotations. The native image sizes are also diverse: in the training split, image widths range from 192 to 1707 pixels and heights from 180 to 1921 pixels, whereas in the validation split, widths range from 186 to 4096 pixels and heights from 242 to 4096 pixels. Across the full dataset, we annotated 10,557 axis-aligned bounding boxes in total, including 4644 cat boxes, 4653 head boxes, and 1260 tail boxes. All images are annotated with axis-aligned bounding boxes for the three categories and split into training and validation sets at the image level. The dataset covered diverse lighting conditions, viewpoints, scenes, and cat appearances, providing broad visual variation for robust part-level detector training.

#### 2.1.2. Video Dataset for Behavior Recognition

For downstream behavior recognition, we constructed a video dataset of 1283 short cat clips collected from unconstrained real-world environments. The videos are primarily recorded by the authors and further supplemented with permissively licensed online footage. Of the 1283 clips, 709 were self-collected and 574 were obtained from publicly licensed online sources, corresponding to 55.3% and 44.7% of the dataset, respectively. This combination increases variation in scene context, camera viewpoints, scale, illumination, and behavioral presentation, making the dataset suitable for evaluating clip-level cat behavior recognition under realistic conditions. Each clip was assigned a single behavior label as defined in Section 2.2.

Because the self-collected footage was acquired through opportunistic random recording rather than a formally logged longitudinal study, we did not rely on exhaustive per-cat identity annotation for dataset partitioning. Instead, to reduce potential data leakage, the dataset was partitioned conservatively before clip extraction at the original-source, raw-video, recording-session, and stable scene-context levels, rather than at the clip level. Accordingly, clips extracted from the same raw video or continuous recording session were always assigned to the same partition, and visually near-duplicate scene contexts were not split across the training, validation, and test sets whenever such continuity could be reliably identified. In addition, the self-collected and permissively licensed online footage were curated from disjoint source pools, and no repeated cat individual across these source pools was known during dataset construction.

The final split contained 969 training clips, 154 validation clips, and 160 test clips. In addition, the union of the training and validation splits (1123 clips in total) was also used at the unlabeled-frame level for detector-robustness assessment, whereas clip-level behavior annotations were used only in the downstream recognition stage. The held-out test split was reserved for final evaluation and was not used in detector robustness analysis or detector selection. We also manually checked for potentially repeated individuals and visually near-duplicate scene contexts across partitions, and handled uncertain cases conservatively during split curation. This split design supported controlled detector analysis and downstream behavior recognition evaluation while minimizing potential data leakage.

### 2.2. Annotation Protocol

This study adopted a two-level annotation protocol: (i) part-level bounding-box annotation for detection and (ii) clip-level single-label annotation for behavior recognition. The protocol was designed to reduce ambiguity and improve annotation consistency through explicit class definitions and deterministic precedence rules.

#### 2.2.1. Part-Level Detection Annotation

**Figure 1:**
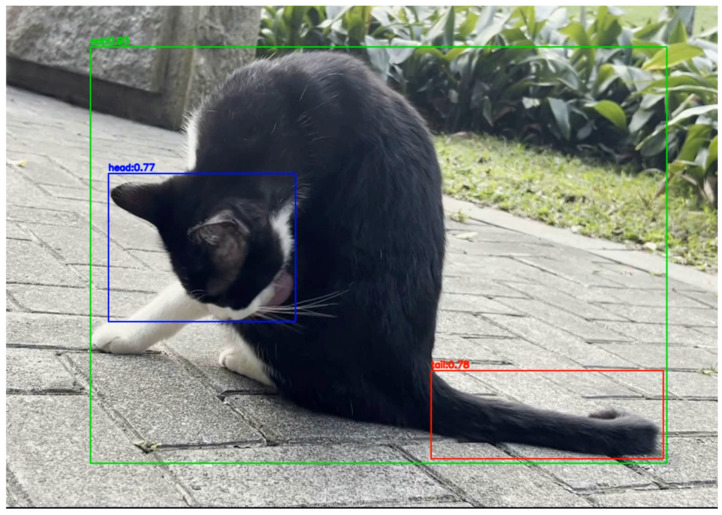
Example of part-level annotation.

All detection images were annotated in standard COCO format with axis-aligned bounding boxes for three categories: cat, head, and tail. Each bounding box was tightly fitted to the visible boundary of the corresponding target. If an anatomical part was fully occluded or not visible, no bounding box was assigned to that part. This visibility-based omission rule kept the annotation protocol consistent with the downstream missing-aware formulation. The three annotated categories were consistently encoded as cat/head/tail = 0/1/2 in detector training. Figure 1 shows an example of the part-level annotation used in this study, where the cat, head, and tail are enclosed by separate axis-aligned bounding boxes.

#### 2.2.2. Behavior Annotation (Clip-Level)

For clip-level behavior recognition, we adopted a compact five-class taxonomy: Rest, Locomotion, Grooming, Active, and Tail-Expressive. This taxonomy was not intended to exhaust the full repertoire of domestic cats, but rather to provide a study-specific, task-oriented aggregation suitable for clip-level video annotation. Detailed feline ethograms contain many fine-grained and context-dependent behaviors, which are difficult to label consistently in short real-world clips. We therefore grouped behaviors into five visually and functionally interpretable dominant states to improve annotation consistency and reduce ambiguity. Specifically, Rest represents low-activity static states such as sitting and lying; Grooming denotes self-directed maintenance behaviors; Locomotion captures sustained body translation; Active refers to intense whole-body movement such as pouncing, jumping, chasing, or rapid turning; and Tail-Expressive isolates clips in which tail motion is the dominant discriminative cue while overall body displacement remains limited [28,29,30,31].

**Figure 2:**
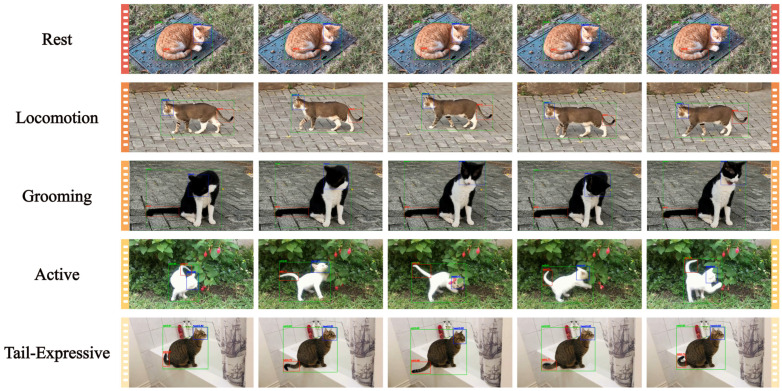
Representative real-video frames illustrating the five behavior categories used for clip-level annotation: Rest, Locomotion, Grooming, Active, and Tail-Expressive.

Short real-world clips often contain mixed or transitional cues. Therefore, we used a single-label scheme with deterministic precedence rules to assign each clip its dominant behavioral state. This design improved annotation consistency and benchmarking clarity while preserving behaviorally meaningful distinctions relevant to welfare-oriented monitoring and video recognition. Within this formulation, consolidating low-activity static postures into Rest helped focus the taxonomy on dynamic and locally expressive cues, especially head- and tail-related motion, which were central to the present pipeline design [28,30,32]. Figure 2 provides representative real-video examples of the five behavior categories used in our clip-level annotation protocol, and Table 1 summarizes the corresponding task-level class definitions.

To assess clip-level annotation reliability, the complete set of 1283 behavior clips was independently reviewed by a second annotator using the same five class definitions and single-label rules. The overall agreement was 97.6% (1252/1283), with a Cohen’s kappa of 0.969 [33]. The 31 disagreements were resolved by joint review according to the predefined annotation rules.

For transparency, the class-wise distribution of the behavior video dataset is summarized in Table 2. The corpus contained 1283 clips in total, including 326 Rest clips, 241 Locomotion clips, 401 Grooming clips, 202 Active clips, and 113 Tail-Expressive clips. All five categories were represented in the training, validation, and test sets, and the split proportions were broadly comparable across partitions. The dataset was designed as a task-oriented benchmark for clip-level cat behavior recognition in unconstrained videos, with an emphasis on realistic variability, conservative split hygiene, and controlled evaluation. Although the dataset was moderate in scale, its size is appropriate for the scope of the present study. The task was formulated as a compact five-class clip-level recognition problem rather than an exhaustive fine-grained ethogram, which helped keep the label space consistent with the available number of annotated clips. In addition, the 1283 videos were collected from heterogeneous real-world sources and were split conservatively by source, raw video, recording session, and stable scene context. Therefore, the reported performance reflects evaluation on held-out videos rather than on randomly separated clips from the same continuous recordings. The Stage-3 recognizer was also intentionally lightweight and trained on frozen detector-derived ROI features, which reduced the number of trainable parameters compared with end-to-end raw-video models and helped control overfitting under the current dataset scale.

#### 2.2.3. Disambiguation and Consistency Rules

Because real-world clips may contain multiple co-occurring cues, we assigned a single label according to the following deterministic precedence order.

1. Primary-activity precedence: If Grooming or Active behavior was clearly present, the clip was labeled accordingly, even if tail motion co-occurs.
2. Locomotion precedence: If sustained walking/running occurred, the clip was labeled as Locomotion, independent of camera panning or following.
3. Tail-dominant expression: If the body was largely stationary but tail motion was persistent and salient, the clip was labeled as Tail-Expressive.
4. Default to Rest: Clips with only minor head motion and without the above behaviors were labeled as Rest.

These rules encoded a consistent operational definition of “dominant behavior” for single-label annotation. To reduce subjectivity, we (i) kept the taxonomy compact, (ii) defined each class by observable cues, and (iii) applied deterministic precedence rules that were used for all clips. This design helped improve annotation consistency across clips and reduces avoidable label ambiguity, which was important for learning reliable temporal patterns from limited labeled video data.

### 2.3. Overall Pipeline

**Figure 3:**
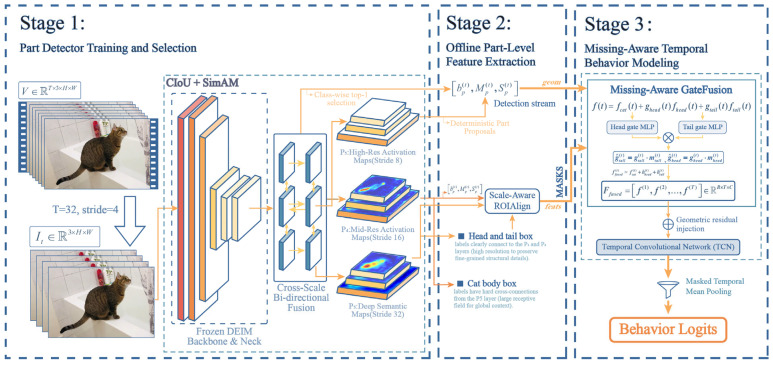
Overall three-stage pipeline of PMTNet.

Figure 3 shows the three-stage pipeline of PMTNet. Given an input cat video, the method first localizes three anatomical parts (cat, head, and tail) frame by frame, then exports part-aligned ROI representations from the selected detector, and finally predicts the clip-level behavior label through missing-aware fusion and temporal modeling.

Stage 1: Part Detector Training and Selection.

We trained a detector based on DETR with Improved Matching (DEIM) to localize three anatomical parts, including the cat body, head, and tail, on individual frames. Multiple detector variants were then compared under a downstream-aware evaluation protocol to identify the model that provided the most reliable part trajectories for subsequent temporal modeling. Details of detector construction and selection are given in Section 2.4.

Stage 2: Offline Part-Level Feature Extraction.

After detector selection, the chosen model was frozen and applied frame by frame to each video clip. For each frame, we retained class-wise Top-1 part detections and exported the corresponding bounding boxes, ROI-aligned appearance features extracted from the frozen detector feature maps, and visibility masks. These cached part-level representations served as the input to the downstream temporal recognizer.

Stage 3: Missing-Aware Temporal Behavior Modeling.

Based on the cached part-level outputs, we constructed sequential cues from both ROI appearance embeddings and explicit geometric motion descriptors. To handle intermittent part missingness, especially for the tail, we used a missing-aware fusion module to aggregate the available part information at each time step, followed by a lightweight temporal encoder for final clip-level behavior classification. The detailed formulation of feature construction, fusion, and temporal modeling is presented in Section 2.5.

We used offline feature caching mainly for efficient and controlled Stage-3 training. This decoupled design avoided repeatedly executing the heavy detector during temporal-model optimization and ensured a shared feature basis for subsequent temporal and fusion ablations. Although training was performed offline, the overall architecture remained feed-forward: during deployment, detection, ROI extraction, and temporal prediction could still be executed sequentially in an online streaming manner. This design made the framework more suitable for practical non-invasive monitoring scenarios where behavior cues had to be be extracted from ordinary videos without attaching sensors to the animal.

### 2.4. Downstream-Aware Part Detector: DEIM Backbone, Candidate Variants, and Selection

#### 2.4.1. DEIM-Based Part Localization and Candidate Construction

Our pipeline required frame-wise localization of three anatomical parts—cat, head, and tail—to support downstream ROI-based temporal behavior modeling. However, cat extremities, especially the tail, frequently exhibit motion blur, strong deformation, and partial occlusion in real-world videos. Standard Detection Transformer (DETR)-style detectors rely on sparse one-to-one assignment and therefore often struggle to maintain stable supervision for such small and highly dynamic parts, which can lead to intermittent missing detections and bounding-box jitter [34].

To address this issue, we adopted DEIM as the detector framework [35]. DEIM introduces two mechanisms that are particularly relevant to our setting. First, Dense O2O (Dense One-to-One Supervision) increases the density of positive supervision during training, which helps the detector pay sustained attention to hard-to-localize extremities. Second, Matchability-Aware Loss (MAL) remains sensitive to low-quality early matches and therefore helps preserve supervision for blurred or partially occluded parts during box refinement. Together, these mechanisms improve detector robustness for highly articulated cat parts.

Our goal, however, was not to perform an exhaustive search over all possible detector architectures, but to identify, under a controlled detector family, the candidate that yielded the most reliable part trajectories for downstream temporal modeling. We therefore constructed a controlled candidate pool within the same DEIM framework. In implementation, all detector candidates were instantiated from the same HGNetv2-L DEIM configuration, implemented from deim_hgnetv2_l_coco.yml. Here, HGNetv2-L denotes the large version of the HGNetv2 convolutional neural network backbone. We varied only the geometric regression objective, including Generalized IoU (GIoU), Distance IoU (DIoU), Complete IoU (CIoU), and Wise IoU v3 (WIoU v3), together with optional lightweight feature-enhancement modules, including the Convolutional Block Attention Module (CBAM) and the Simple Parameter-Free Attention Module (SimAM) [36,37,38,39,40,41]. This controlled design made the comparison easier to interpret. If the backbone or detection paradigm were changed, differences in downstream performance could also come from model capacity, feature hierarchy, optimization behavior, or assignment dynamics. By keeping the detector family, backbone, and training recipe fixed, we isolated a practically relevant question for the present pipeline: which detection-side design within a common part-localization framework provided the most usable trajectories for downstream behavior recognition. In this way, differences in downstream temporal robustness could be attributed primarily to the controlled IoU-family objectives and lightweight enhancement modules rather than to unrelated training changes. All candidate detectors were trained under the same implementation-faithful schedule: the input size was fixed at 640, the total training length was 70 epochs, policy.epoch = [4, 29, 50], mixup_epochs = [4, 29], flat_epoch = 29, and no_aug_epoch = 8 were kept unchanged across runs. In the DEIM detector-training stage, image-level data augmentation was applied to the 4000 training images used for cat/head/tail part detection, while the 500 validation images was used only for evaluation. Following the same DEIM training recipe for all detector candidates, the augmentation pipeline included Mosaic, Random Photometric Distort, Random Zoom Out, Random IoU Crop, Random Horizontal Flip, and resizing to 640 × 640. MixUp was also enabled during the scheduled early training phase defined by mixup_epochs = [4, 29]. These augmentation settings, together with the input size and training schedule, were kept identical for all detector variants, including GIoU, DIoU, WIoU v3, CIoU, CIoU + CBAM, and CIoU + SimAM, to ensure a fair comparison among detector-side design choices. After detector training, the best checkpoint of each candidate was used for video-domain robustness evaluation and subsequent Stage-3 feature export. In addition, stop_epoch = 50 was used only as an internal stage-transition boundary rather than as early stopping. The best checkpoint of each candidate was then exported for subsequent video-based robustness evaluation and offline Stage-3 feature extraction. Cross-family comparison was treated only as an informative reference. The YOLOv8-m result is reported in Section 3.1, and broader video recognition baselines are reported in Section 3.3.

We restricted the detector comparison to variants within the same DEIM family for both methodological and practical reasons. Methodologically, this controlled setting kept the backbone, assignment strategy, training schedule, and detector capacity fixed, allowing the effect of IoU-family losses and lightweight enhancement modules on downstream temporal usability to be isolated. Practically, using one detector family also limited computational cost and avoided introducing cross-architecture differences that would have been difficult to attribute under the current dataset scale. Therefore, cross-family detector comparison was treated as an informative reference rather than the main focus of detector selection.

#### 2.4.2. Formulation of Temporal Robustness Metrics

To quantify the robustness of part-level detection in videos, we evaluated each detector on 1123 real-world clips drawn from the training and validation splits of the behavior-recognition dataset. This analysis was performed only at the unlabeled-frame level to characterize detector behavior in the target video domain; the corresponding clips were not used to optimize detector parameters, and the held-out test split was excluded. Let c denote a clip index, and let $I^{\left(c\right)}={\left\{I_{t}^{\left(c\right)}\right\}}_{t=1}^{T_{c}}$ denote the sampled frame sequence of clip c after fixed-stride subsampling, where $T_{c}$ is the number of sampled frames. With this protocol, the evaluation comprised 47,003 sampled frames in total. For each sampled frame $I_{t}^{\left(c\right)}$ and part $p\in \left\{cat,head,tail\right\}$, we ran the detector and retained the class-wise Top-1 prediction with confidence score $s_{p,t}^{\left(c\right)}$. Let $b_{p,t}^{\left(c\right)}$ denote the retained box and let τ denote the confidence threshold for Top-1 part retention (τ=0.3 in our implementation). If $s_{p,t}^{\left(c\right)}<\tau$, or if $b_{p,t}^{\left(c\right)}$ is geometrically invalid (e.g., non-positive area), that part was marked as missing at frame t. This protocol evaluated two aspects of detector quality. The first was frame-level detection accuracy. The second was whether the detected body parts remained available and stable across video frames.

We grouped the temporal robustness metrics into three categories: detection availability, absolute temporal stability, and cat-relative temporal stability. For efficiency, frames were uniformly subsampled with a fixed stride. Based on the retained Top-1 predictions, we defined a binary validity indicator $v_{p,t}^{\left(c\right)}$ for each clip, part, and sampled frame, where $v_{p,t}^{\left(c\right)}=1$ if the retained detection for part p at frame t of clip c existed, exceeded the confidence threshold τ, and formed a valid box, and $v_{p,t}^{\left(c\right)}=0$ otherwise. Detection availability measures whether each part can be reliably retained over time, using missing rate (MissRate), valid rate (ValidRate), and mean confidence (MeanConf).

(1) $$valid\_frac^{\left(p\right)}=\frac{1}{T}\sum_{t=1}^{T}v_{t}^{\left(p\right)},$$

(2) $$missing\_rate^{\left(p\right)}=1-valid\_frac^{\left(p\right)},$$

The mean confidence score was averaged over valid frames only:

(3) $$mean\_score^{\left(p\right)}=\frac{\sum_{t=1}^{T}v_{t}^{\left(p\right)}s_{t}^{\left(p\right)}}{\sum_{t=1}^{T}v_{t}^{\left(p\right)}},$$

where $s_{t}^{\left(p\right)}$ denotes the confidence of the retained Top-1 detection for part p at frame t. These quantities describe whether a detector can maintain continuous and confident part availability over time.

Absolute temporal stability was computed over consecutive valid detections, with missing frames skipped. It describes how smoothly the retained part box changes in overlap, position, and scale. Let $b_{p,t}^{\left(c\right)}$ be the retained bounding box for part p at frame t of clip c, and let $V_{p}^{\left(c\right)}=\left\{t|v_{p,t}^{\left(c\right)}=1\right\}$ denote the ordered index set of valid frames for that part. We defined $N_{p}^{\left(c\right)}$ as the number of valid consecutive pairs induced by $V_{p}^{\left(c\right)}$ and computed three absolute stability metrics. First, the IoU jitter was defined as:

(4) $$jitter_{iou}\left(p\right)=1-\frac{1}{K}\sum_{k=1}^{K}I\;oU\left(B_{t}^{\left(p\right)},B_{t_{k-1}}^{\left(p\right)}\right),$$

This quantity measures frame-to-frame overlap inconsistency, with smaller values indicating higher temporal stability. For easier interpretation, we also reported the equivalent mean IoU stability:

(5) $$meanIoU\left(p\right)=1-jitter_{iou}\left(p\right),$$

so that larger values indicated better stability.

Second, we measured center jitter using normalized center displacement. Let $c\left(B\right)$ denote the box center, and let $w\left(B\right)$ and $h\left(B\right)$ denote the width and height of box B. We normalized the displacement by the geometric mean size of the previous valid box:

(6) $$jitter_{center}\left(p\right)=\frac{1}{K}\sum_{k=1}^{K}\frac{\|c(B_{t_{k}}^{\left(p\right)})-c(B_{t_{k-1}}^{\left(p\right)}){\|}_{2}}{\sqrt{w(B_{t_{k-1}}^{\left(p\right)})h(B_{t_{k-1}}^{\left(p\right)})}},$$

This normalization reduced the scale dependence of raw center displacement and made the metric more comparable across parts of different sizes. Third, we defined scale jitter in log space to quantify multiplicative changes in box size:

(7) $$jitter_{scale}\left(p\right)=\frac{1}{K}\sum_{k=1}^{K}\left(\left|log\frac{w(B_{t_{k}}^{\left(p\right)})}{w(B_{t_{k-1}}^{\left(p\right)})}\right|+\left|log\frac{h(B_{t_{k}}^{\left(p\right)})}{h(B_{t_{k-1}}^{\left(p\right)})}\right|\right),$$

Intuitively, IoU jitter reflects changes in box overlap, center jitter reflects abrupt position shifts, and scale jitter reflects sudden changes in box size.

This metric is sensitive to abrupt width and height fluctuations, which often arise from unstable localization or rapid deformation.

Cat-relative temporal stability was used for the head and tail to reduce the influence of camera motion and whole-body translation. By expressing each part box in the coordinate system of the detected cat box, these metrics focus on local stability relative to the body. Let $B_{t}^{\left(cat\right)}$ denote the detected cat box at frame t, and let $B_{t}^{\left(p\right)}$ denote the detected box of part p at frame t. For a part box $B_{t}^{\left(p\right)}$, we defined its cat-relative normalized version $Normalize(B_{t}^{\left(p\right)};B_{t}^{\left(cat\right)})$, where normalization subtracted the top-left corner of the cat box and divided by the cat-box width and height. Relative metrics were computed only on frames where both the part and the cat were valid. Let ${\left\{t_{k}\right\}}_{k=1}^{{N_{p}}^{\prime }}$ denote the ordered frame indices satisfying this constraint for part p, and let ${N_{p}}^{\prime }={N_{p}}^{\prime }-1$ be the number of valid consecutive pairs. We then defined the relative IoU jitter as:

(8) $$rel\_jitter_{iou}\left(p\right)=1-\frac{1}{K^{\prime }}\sum IoU\left({\hat{B}}_{t_{k}}^{\left(p\right)},{\hat{B}}_{t_{k-1}}^{\left(p\right)}\right),$$

and the relative center jitter as:

(9) $$rel\_jitter_{center}\left(p\right)=\frac{1}{K^{\prime }}\sum {\left\|\hat{c}({\hat{B}}_{t_{k}}^{\left(p\right)})-\hat{c}\left({\hat{B}}_{t_{k-1}}^{\left(p\right)}\right)\right\|}_{2},$$

These relative metrics better reflected whether head and tail detections remained stable with respect to the cat body, rather than being dominated by global scene motion. Together, these three metric groups characterized whether part evidence is available, temporally smooth, and stable relative to the cat body, forming the basis of the downstream-aware detector comparison in Section 3.1.

#### 2.4.3. Part-Level Feature Extraction and Offline Caching

During Stage 2, the part detector used for feature extraction was finalized after the two-step selection procedure reported in Section 3.1 and Section 3.2.1: candidate detectors were first narrowed by the temporal robustness protocol in Section 2.4.2, and the final detector was then selected by closed-loop downstream behavior-recognition performance. For each sampled frame in a clip, the frozen DEIM detector produced a set of query-level predictions. To obtain a deterministic part-level representation, we performed class-wise Top-1 selection for each semantic part $p\in \left\{cat,head,tail\right\}$. Specifically, among all detector queries, we retained the highest-confidence proposal for each part independently:

(10) $$q_{t}^{\left(p\right)}=arg\;\underset{q}{max}\;s_{t,q}^{\left(p\right)},$$

where $s_{t,q}^{\left(p\right)}$ denotes the classification score of query q for part p at frame t. We performed this per-class selection independently across all queries, rather than taking a per-query argmax, so that the most confident proposal for each anatomical region was preserved.

Let $b_{t}^{\left(p\right)}\in {\mathbb{R}}^{4}$ denote the selected bounding box and $c_{t}^{\left(p\right)}\in \left[0,1\right]$ denote its confidence score. A selected proposal was considered valid only when two conditions were satisfied simultaneously: its confidence exceeded the predefined threshold τ, and its box geometry was valid for ROI extraction. We therefore defined a binary visibility mask

(11) $$m_{t}^{\left(p\right)}=\left\{\begin{array}{l} 1,\;\;\;c_{t}^{\left(p\right)}>\tau \;and\;b_{t}^{\left(p\right)}\;is\;valid\\ 0,\;\;\;otherwise. \end{array}\right.,$$

When $m_{t}^{\left(p\right)}=0$, the corresponding confidence was set to zero. The procedure yielded three temporally aligned sequences for each clip: bounding boxes $b_{t}^{\left(p\right)}$, visibility masks $m_{t}^{\left(p\right)}$, and confidence scores $c_{t}^{\left(p\right)}$. By storing boxes, masks, and scores, the representation recorded when a part is missing. This is particularly important for the tail, which is often affected by self-occlusion, motion blur, or out-of-frame movement.

Using the frozen detector feature maps, we then applied Region of Interest Align (ROIAlign) to each valid box to extract part-aligned appearance embeddings [42]. In implementation, the confidence threshold for Top-1 part retention was fixed to τ=0.3. ROIAlign was performed on multi-level detector feature maps with strides (8,16,32), where the cat body was extracted from level 1 and the head and tail were extracted from level 0, reflecting the different spatial scales of the three parts. Each valid RoI was aligned to a 7×7 feature patch with sampling ratio 2 and aligned sampling enabled, followed by average pooling to obtain a 256-dimensional appearance embedding. Importantly, these appearance features were extracted from the detector’s internal feature representations rather than from raw cropped image patches, so that the downstream recognizer received part-aligned visual descriptors that remained consistent with the frozen detection backbone. The final cached representation for each part and frame was therefore

(12) $$z_{t}^{p}=\left(f_{t}^{p},m_{t}^{p},s_{t}^{p},b_{t}^{p}\right),$$

where $\;f_{t}^{p}\;$ denotes the ROI-aligned appearance embedding, $m_{t}^{p}$ the binary validity mask, $s_{t}^{p}$ the confidence score, and $b_{t}^{p}\;$ the selected Top-1 box. These cached representations were stored to disk and reused in Stage 3. The decoupled design not only reduced repeated execution of the heavy detection backbone during temporal-model training, but also ensured that subsequent temporal, fusion, and readout comparisons were conducted on a shared detector-derived feature basis. In addition, invalid or low-confidence boxes were excluded from ROI feature extraction, which reduced unnecessary background contamination in the downstream recognizer.

### 2.5. Stage 3: Missing-Aware Temporal Behavior Modeling

#### 2.5.1. Stage-3 Inputs and Offline Clip Construction

Stage 3 operated on cached part-level outputs exported by the frozen DEIM detector, rather than directly optimizing on raw video frames. Therefore, conventional online spatial augmentation of raw frames, such as random cropping, horizontal flipping, color jittering, or other photometric transformations, was not applied during Stage-3 behavior-recognition training. Instead, the Stage-3 recognizer sas trained on offline cached representations, including cat/head/tail ROI appearance features, visibility masks, confidence scores, and geometric motion descriptors.

Temporal variation in Stage 3 was introduced through a limited cached-clip sampling strategy. Given a target temporal length T, stride s, and decoded video length L, we defined the feasible start range as $\left[0,max(0,L-\left(\left(T-1\right)s+1\right)\right)]$. For each video, three deterministic clips were exported offline using normalized offsets $\left\{0.0,0.5,1.0\right\}$ over the feasible temporal range, corresponding to the start, middle, and end clips. In the default setting, all videos were decoded or resampled to 30 fps before clip construction. We used T = 32 and stride = 4 for each model input, corresponding to an effective temporal span of approximately 4.1 s. During training, one cached clip was randomly selected for each video at each epoch, whereas validation and testing used all cached clips and averaged their logits to obtain the final video-level prediction.

For short videos where the available decoded frames were insufficient to cover the required temporal span, the implementation did not repeat the last frame and did not use circular wrap-around. Instead, it performed adaptive evenly spaced resampling over the available decoded frames, so that every sample still contained exactly T frames while avoiding artificial motion artifacts. In the offline training pipeline, one cached clip was randomly selected for each video at every epoch. During validation and testing, all cached clips were evaluated and their logits were averaged to form the final video-level prediction. This design preserved the original temporal-cropping rationale while making Stage-3 optimization reproducible and computationally efficient. For single-clip debugging runs, we set K=1 and used the center clip by default.

#### 2.5.2. Explicit Geometric Motion Cue Construction

In addition to the cached ROI-aligned appearance embeddings, we constructed an explicit low-dimensional geometric motion descriptor from the frame-wise Top-1 part boxes. Let $B_{t}^{cat},B_{t}^{head}$, and $B_{t}^{tail}$ denote the selected boxes at frame t. Each box was converted to a center-size representation $\left(c_{x},c_{y},w,h\right).$ For the cat box, all four quantities were normalized by the image width and height. For the head and tail boxes, the same four quantities were expressed relative to the detected cat box, i.e., their centers were normalized in the cat coordinate system and their widths and heights were divided by the cat-box width and height. Accordingly, the per-frame static geometry vector was

(13) $$q_{t}=\left[q_{t}^{cat},q_{t}^{head},q_{t}^{tail}\right]\in {\mathbb{R}}^{12},$$

where each part contributed four dimensions corresponding to normalized center x, center y, width, and height. We further computed first-order temporal differences

(14) $$\Delta q_{t}=q_{t}-q_{t-1}\in {\mathbb{R}}^{12},$$

which encoded frame-to-frame geometric motion trends. Missing parts were zeroed consistently with the ROI validity mask, and motion differences were retained only for consecutive valid steps. The final geometric motion descriptor was therefore

(15) $$g_{t}=\left[q_{t},\Delta q_{t}\right]\in {\mathbb{R}}^{24},$$

In this way, the descriptor explicitly encoded both global body geometry and local part geometry relative to the cat body, together with their short-term temporal variation. For example, when the cat body remained largely stationary but the tail swung laterally, the cat-related dimensions changed only slightly, whereas the tail-relative center coordinates and their temporal differences varied markedly, providing an explicit cue for Tail-Expressive clips. The resulting 24-dimensional descriptor was then projected to the same 256-dimensional feature space as the appearance stream by a two-layer multilayer perceptron (MLP) before fusion.

**Figure 4:**
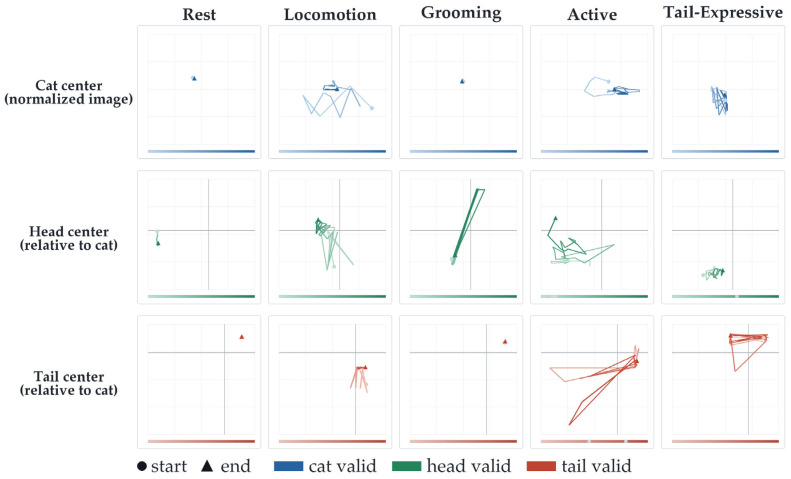
Detector-derived part trajectories used to construct the geometric motion descriptor. The upper row shows cat-body center trajectories in normalized image coordinates, whereas the middle and lower rows show head and tail center trajectories relative to the detected cat box. Color gradients indicate temporal order within each part stream, and circles and triangles denote the start and end of each sampled clip, respectively. The colored bars below each panel indicate valid sampled frames for the corresponding part stream; gray gaps, when present, indicate missing detections.

To make the geometric motion descriptor more interpretable, we visualize representative detector-derived part trajectories in Figure 4. These trajectories were computed directly from the cached Top-1 cat, head, and tail detections used for Stage-3 modeling. The cat-body center trajectory is shown in normalized image coordinates, whereas the head and tail center trajectories are shown relative to the detected cat box, consistent with the coordinate normalization used in the proposed geometric motion descriptor. As shown in Figure 4, Locomotion exhibits more evident global body displacement, while Grooming and Tail-Expressive show more pronounced local relative motion of the head and tail, respectively. This visualization provides an intuitive view of how the descriptor represents both global body movement and local part dynamics.

#### 2.5.3. Missing-Aware Fusion Mechanism

At each time step, the recognizer received three part-aligned appearance features together with the projected geometric motion embedding. Before temporal modeling, the three part streams were fused into a single missing-aware per-frame representation. We considered two fusion strategies.

As a non-adaptive baseline, ConcatFusion first masks missing parts and then concatenates the cat, head, and tail features, followed by a linear projection back to the hidden dimension:

(16) $${\hat{f}}_{t}^{concat}=W_{concat}\left[m_{t}^{\left(cat\right)}f_{t}^{\left(cat\right)}\|\;m_{t}^{\left(head\right)}f_{t}^{\left(head\right)}\|\;m_{t}^{\left(tail\right)}f_{t}^{\left(tail\right)}\right],$$

This baseline uses the same inputs and the same missingness handling as the gated version, differing only in whether the contribution of each part is adaptively controlled.

Our main model adopts GateFusion, a missing-aware gating mechanism that adaptively controls the contribution of the head and tail at each frame. Let $\alpha_{t}^{\left(head\right)}$ and $\alpha_{t}^{\left(tail\right)}$ denote the learned gates. The fused appearance feature is defined as:

(17) $${\hat{f}}_{t}^{gate}=f_{t}^{\left(cat\right)}+m_{t}^{\left(head\right)}\alpha_{t}^{\left(head\right)}⊙f_{t}^{\left(head\right)}+m_{t}^{\left(tail\right)}\alpha_{t}^{\left(tail\right)}⊙f_{t}^{\left(tail\right)},$$

The gates are predicted by a lightweight MLP conditioned on per-frame cues, including the cat feature, the corresponding part feature, and the visibility mask. In our implementation, the input to each gate is a 513-dimensional vector formed by concatenating the 256-dimensional cat feature, the 256-dimensional part feature, and a 1-dimensional visibility mask. Each gate was predicted by a two-layer multilayer perceptron with hidden dimension 256:

(18) $$g=W_{2}\sigma \left(W_{1}x+b_{1}\right)+b_{2},$$

where $x\in {\mathbb{R}}^{513}$ denotes the gate input, $\sigma \left(\cdot \right)$ is the ReLU activation, $W_{1}\in {\mathbb{R}}^{256\times 513}$, and $W_{2}\in {\mathbb{R}}^{1\times 256}$. To enforce missing-aware fusion, the predicted gate was multiplied by the corresponding binary visibility mask, so that missing parts contribute dzero. The projected geometric motion embedding was then merged with the fused appearance stream to form the final per-frame input to the temporal module.

#### 2.5.4. Temporal Sequence Encoder

Given the fused per-frame representation $x_{t}\in {\mathbb{R}}^{d}$ at time step t, which integrates ROI appearance features and projected geometric motion cues, we employed a lightweight temporal sequence encoder to model clip-level behavioral dynamics. Let $X=\left[x_{1},\ldots ,x_{T}\right]\in {\mathbb{R}}^{T\times d}$ denote the input sequence, and let $u=\left[u_{1},\ldots ,u_{T}\right]$ denote the corresponding temporal validity mask, where $u_{t}\in \left\{0,1\right\}$ indicates whether the frame remains temporally valid after missing-aware preprocessing. We investigated two complementary temporal encoders, namely a Temporal Convolutional Network (TCN) and a Gated Recurrent Unit (GRU), under the same input representation and downstream classifier setting for fair comparison [43,44].

For the TCN, temporal evolution was modeled by applying one-dimensional convolutions along the time axis, enabling the network to aggregate local motion patterns and short-range part interactions in parallel. Given the input sequence X, the TCN produces a hidden sequence

(19) $$H^{TCN}=TCN\left(X\right),$$

(20) $$H^{TCN}\in {\mathbb{R}}^{T\times d},$$

This design is simple and computationally efficient, and is well suited for capturing local temporal continuity in part-level motion cues.

As an alternative sequential encoder, we employed a GRU to recursively update the hidden state over time, allowing the model to accumulate behavior-relevant evidence across the clip. For each time step t, the GRU updates its hidden state as

(21) $$h_{t}^{GRU}=GRU\left(x_{t},h_{t-1}^{GRU}\right),$$

Compared with the TCN, the GRU preserves stronger sequential ordering and can better encode temporally asymmetric behavior transitions.

For both temporal encoders, the output is a temporally structured hidden representation that is subsequently aggregated into a clip-level feature using the mask-aware readout strategies described in Section 2.5.5. Unless otherwise specified, the temporal encoder type, TCN or GRU, was treated as an ablation factor in Stage-3 experiments, while all other components were kept identical.

We selected TCN and GRU as the primary temporal encoders because they provided two complementary but lightweight ways to model short clip-level dynamics under the current dataset scale. The TCN captures local temporal patterns through parallel temporal convolutions, whereas the GRU models sequential dependency through recurrent state updates. Compared with larger transformer-based temporal models, both encoders have fewer trainable parameters and are less likely to overfit when trained on frozen ROI-level features from a moderate-sized behavior dataset. This choice also kept the Stage-3 ablation focused on whether local convolutional or recurrent temporal modeling was more suitable for missing-aware part streams.

Transformer-based temporal encoders were not included as Stage-3 alternatives in the main ablation because the focus of this study was on lightweight missing-aware fusion over detector-derived part streams rather than on scaling temporal model capacity. Instead, transformer-based video architectures were considered at the baseline level in Section 3.3 through full-frame TimeSformer and VideoMAE comparisons.

#### 2.5.5. Temporal Readout and Sequence Aggregation

After temporal encoding, we obtained a hidden sequence $H={\left\{h_{t}\right\}}_{t=1}^{T}$, where each $h_{t}$ summarizes behavior-relevant information at time step t. Because the final label was defined at the clip level, the hidden sequence had to be be further aggregated into a single fixed-dimensional representation. To ensure robustness under missing parts and variable valid temporal support, this aggregation is performed in a mask-aware manner using the temporal validity mask u.

We investigated three aggregation strategies. For last-step aggregation, we used the hidden state of the last valid time step:

(22) $$h_{clip}^{last}=h_{t^{*}},$$

(23) $$t^{*}=\max\{t\;|\;u_{t}=1\},$$

This strategy preserved sequential causality and was particularly natural for recurrent encoders such as the GRU.

For mean aggregation, we computed a masked temporal average over valid steps:

(24) $$h_{clip}^{mean}=\frac{\sum_{t=1}^{T}u_{t}\;h_{t}}{\sum_{t=1}^{T}u_{t}+\varepsilon },$$

where ε is a small constant for numerical stability. This aggregation is parameter-free and encourages the classifier to rely on the overall temporal tendency of the clip rather than a single instant.

For attention aggregation, we learned a temporal weighting over valid steps:

(25) $$e_{t}=w_{a}^{⊤}tanh(W_{a}h_{t}),$$

(26) $$\alpha_{t}=\frac{u_{t}exp(e_{t})}{\sum_{j=1}^{T}u_{j}exp(e_{j})},$$

(27) $$h_{clip}^{attn}=\sum_{t=1}^{T}\alpha_{t}h_{t},$$

Compared with last-step and mean aggregation, attention aggregation allowed the model to emphasize the most informative moments while suppressing uninformative or ambiguous temporal steps. The aggregated clip representation was then fed into a linear classifier to produce the final behavior logits. In our experiments, sequence aggregation was treated as an independent ablation dimension.

#### 2.5.6. Optimization Objective

Let $z_{i}$ denote the logit vector predicted for the i-th training clip, let $y_{i}$ be the ground-truth class label, and let $p_{i,c}$ denote the posterior probability for class c. To mitigate class imbalance in the behavior dataset, we optimized the Stage-3 recognizer with a class-weighted cross-entropy loss [45]:

(28) $$L=-\frac{1}{N}\sum_{i=1}^{N}w_{y_{i}}\log p_{i,y_{i}},$$

where N is the number of training clips in the current split and $w_{y_{i}}$ is the weight assigned to the ground-truth class of sample  i.

Let $n_{c}$ denote the number of training clips belonging to class c. The class weights were computed from the training split only using inverse-frequency reweighting:

(29) $$w_{c}\propto \frac{1}{n_{c}},$$

so that under-represented classes received larger optimization weights, while frequent classes did not dominate the training gradients. This design was appropriate for our five-class behavior setting, where the class frequencies ere not fully balanced across the training split. As a result, the weighted loss improved optimization fairness across categories and reduced bias toward high-frequency classes. The weighted objective therefore complemented our use of Macro-F1 as the main checkpoint-selection criterion, which emphasized balanced recognition quality across all behavior classes. This weighting was particularly relevant for the Tail-Expressive class, which had the smallest number of training clips. However, the weighted loss was intended to reduce optimization bias rather than to fully compensate for limited class diversity; therefore, performance on this class was interpreted together with class-wise recall and the dataset limitations discussed above.

#### 2.5.7. Evaluation Metrics and Checkpoint Selection

For Stage-3 behavior recognition, we evaluated clip-level classification using Top-1 Accuracy and Macro-F1. Unless otherwise specified, validation and testing used multi-clip inference. For each video, we evaluated all K cached clips under the same setting, obtained the clip logits $z_{k}$, and averaged them:

(30) $$\overset{-}{z}=\frac{1}{K}\sum_{k=1}^{K}z_{k},$$

Metrics were then computed at the video level using the prediction induced by $\overset{-}{z}$. In the default setting, K=3, corresponding to the start, middle, and end clips. In the offline training implementation, the training split also stored K deterministic cached clips per video, and one of them was randomly selected at each epoch rather than decoding a new random crop online. For single-clip debugging runs, K=1, and the center clip was used by default.

Top-1 Accuracy was defined in the standard way by comparing the predicted class with the ground-truth label for each video. For class c, let $TP_{c}$, $FP_{c}$, and $FN_{c}$ denote the numbers of true positives, false positives, and false negatives, respectively. The per-class Precision and Recall were defined as:

(31) $$Precision_{c}=\frac{TP_{c}}{\left(TP_{c}+FP_{c}+\varepsilon \right)},$$

(32) $$Recall_{c}=\frac{TP_{c}}{\left(TP_{c}+FN_{c}+\varepsilon \right)},$$

Here ϵ is a small constant to avoid division by zero. The per-class F1 score was then computed as:

(33) $$F1_{c}=\frac{2TP_{c}}{2TP_{c}+FP_{c}+FN_{c}+\epsilon },$$

and Macro-F1 averaged over all classes:

(34) $$Macro-F1=\frac{1}{C}\sum_{c=1}^{C}F\;1_{c},$$

During training, we logged both validation Accuracy and validation Macro-F1 at every epoch. The best Stage-3 checkpoint was selected by the highest validation Macro-F1. We used Macro-F1 as the main checkpoint-selection criterion because it gives equal importance to all behavior categories and therefore better reflects balanced recognition quality under the class-imbalanced setting of this dataset. By contrast, Accuracy served as a complementary overall measure of instance-level correctness.

After model development and checkpoint selection had been completed on the training/validation splits, the held-out test split was used for final evaluation under a fixed protocol. The test protocol matched validation: all cached clips for each video were evaluated and their logits were averaged. We report Top-1 Accuracy, Macro-F1, and per-class Precision, Recall, and F1 to reflect both overall and class-wise recognition quality. In addition to the main final result, we also report several representative component-wise comparisons under the same fixed held-out evaluation protocol to provide a complete view of the finalized framework.

To assess run-to-run variability, selected Stage-3 settings were repeated with three independent random seeds, and the corresponding results are reported as mean ± standard deviation where available. Other component-wise comparisons were conducted under the same fixed training/validation/test split and evaluation protocol, with checkpoint selection based on validation Macro-F1. We did not perform formal statistical significance testing for all pairwise comparisons; therefore, small numerical differences are interpreted cautiously and mainly used to support component-level trends rather than definitive statistical superiority.

### 2.6. Baseline Models and Comparison Protocol

To position PMTNet against representative full-frame video baselines, we used two types of baselines in this study. Section 3.2.2 and Section 3.2.4 provided structurally matched ROI-based baselines within the same detector-driven recognition pipeline, namely, a simple single-stream Cat baseline and a strong non-adaptive multi-part ConcatFusion baseline. In Section 3.3, we compared PMTNet with representative full-frame video recognition models, namely, SlowFast [9], TimeSformer [46], and VideoMAE [11]. This distinction was important because our goal was not only to compare PMTNet against standard end-to-end video backbones, but also to test whether the proposed structured part-centric design provided benefits beyond simpler ROI-based alternatives.

To provide a controlled and implementation-faithful comparison, all global baselines were initialized from their respective official Kinetics-400 pre-trained checkpoints and fine-tuned under the same training/validation/test split, with model selection based on validation set performance [47]. For each baseline, we followed the corresponding official recipe as closely as possible and made limited validation-based adjustments when necessary to obtain a stable training configuration. These checks were intended to avoid clearly under-trained baseline results rather than to perform an exhaustive architecture-specific hyperparameter search. SlowFast follows the official 4 × 16 × 1 configuration with an R50 backbone, whereas TimeSformer follows the official 8 × 32 × 1 configuration under the ViT-Base/Patch16/224 setting. For TimeSformer, we reported both the official stochastic gradient descent (SGD) default and an AdamW optimizer variant because this baseline was empirically sensitive to optimization configuration on the present moderate-scale cat dataset; in this sense, the AdamW result should be regarded as the stronger primary TimeSformer comparator, whereas the SGD result was retained mainly to expose optimization sensitivity. VideoMAE-Base was likewise initialized from its official pre-trained weights and fully fine-tuned under the same protocol. Standard multi-view inference was adopted during evaluation, and we reported Top-1 Accuracy, Macro-F1, and class-wise Recall for all methods. The final PMTNet configuration was evaluated under the same held-out protocol.

We report the main training settings of the full-frame video baselines in Table 3 for reproducibility. Since these models follow different official training recipes, their optimizer, learning rate, batch size, and input settings were not forced to be identical. All methods were evaluated on the same held-out test split using the same metrics. We did not conduct an exhaustive hyperparameter search for every baseline. Parameters not explicitly overridden in the training script followed the default settings of the corresponding framework.

## 3. Results

### 3.1. Stage-2 Detector Robustness and Candidate Narrowing

**Figure 5:**
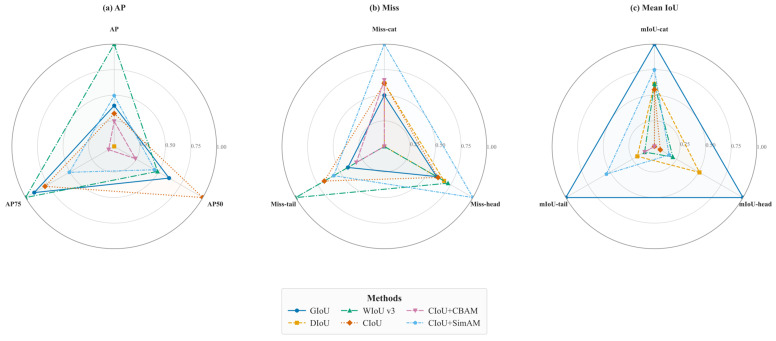
Radar-chart comparison of detector candidates. All axes are normalized so that larger values indicate better performance, including the Miss metrics after inverse normalization.

To bridge the gap between frame-level detection and sequence-level behavior recognition, we evaluated candidate detectors using the downstream-aware protocol defined in Section 2.4.2. Specifically, we compared several IoU-based variants, including GIoU, DIoU, WIoU v3, and CIoU, together with lightweight attention extensions such as CIoU + CBAM and CIoU + SimAM, on the unlabeled training + validation subset of the behavior-recognition dataset, comprising 1123 real-world cat clips in total. With stride-based frame sampling, this evaluation protocol yielded 47,003 assessed frames in total. Importantly, detector training was conducted only on the image-based part-detection dataset, while this video subset was used solely to characterize frame-level part-localization continuity and spatial stability, rather than video-level behavior labels; the held-out test split was reserved for final evaluation only. The resulting static image-domain precision and dynamic video-domain robustness metrics are summarized in Table 4, and their multi-dimensional trade-offs are further visualized in Figure 5.

For downstream behavior recognition, we placed particular emphasis on part missing rates and temporal stability metrics rather than on static AP alone. Missing rates directly reflected whether body, head, and tail evidence remained available across video frames, while mean IoU and relative mean IoU described the stability of retained trajectories. These metrics were therefore most relevant for selecting detectors used in the subsequent temporal recognition stage.

Although all variants provide competitive static detection baselines, Table 4 shows that their downstream-relevant behavior differs more clearly in part missingness and trajectory stability. As shown by the distinct radar-chart profiles in Figure 5, the candidates exhibit a clear multi-dimensional trade-off among strict localization, missingness, and temporal stability. In this figure, larger values on localization and stability-related axes indicate better performance, whereas lower missing rates are preferred and should therefore be interpreted inversely. These differences support our central observation that selecting a detector solely according to static mAP is insufficient for downstream video applications.

Rather than making a premature decision from image-domain detection metrics alone, we used the video-domain robustness analysis to narrow the candidate pool and retain three representative detectors for downstream closed-loop comparison in Stage 3. This radar-chart analysis was used for candidate narrowing rather than final detector adoption, because no single method dominated all axes simultaneously: WIoU v3 was favored for strict localization and low tail missingness, CIoU + SimAM for very low cat/head missingness, and GIoU as a stability-oriented reference.

WIoU v3: retained for its strong strict localization performance, achieving the best AP75 among the compared candidates and the lowest tail missing rate (21.99%), which was beneficial for preserving reliable tail trajectories in downstream ROI-sequence modeling.

CIoU + SimAM: retained for its strong robustness to subject tracking loss, yielding very low missing rates for the cat body and head (0.04% and 6.89%, respectively).

GIoU: retained as a stability-oriented reference candidate, providing the highest mean IoU for the cat body among the DEIM variants (93.47%) and representing a more compact localization regime.

Other intermediate variants, including DIoU, CIoU, and CIoU + CBAM, provided less favorable trade-offs without dominating any specific robustness dimension and were therefore excluded. The three retained candidates were then carried forward to Stage 3 for closed-loop downstream comparison, while the finalized detector setting was subsequently used for the remaining temporal component studies.

YOLOv8-m provided an informative reference: it achieved a relatively high relative mean IoU for the tail (87.93%) but also showed a much higher tail-missing rate (37.25%). This contrast indicates that favorable overlap on retained frames did not necessarily correspond to continuous part availability in videos. Overall, this stage characterized detector candidates from a video-domain robustness perspective and narrowed the candidate pool for subsequent closed-loop verification; it did not use video-level behavior labels to optimize the detector itself.

### 3.2. Stage-3 Ablation Overview

To provide a compact view of the finalized Stage-3 framework, we reported component-wise comparisons over detector variant, part-wise input composition, temporal sequence encoder, fusion strategy, and temporal readout. Unless otherwise specified, all comparisons in this section were evaluated on the same held-out test split under the same protocol. During model development, the main Stage-3 comparisons were repeated in multiple runs, and the relative trends were found to be largely consistent. To keep the main tables concise, we reported detailed mean ± standard deviation only for the temporal readout comparison, where the best-performing configuration was finalized. For the remaining ablations, the tables present representative results under the fixed split and the same checkpoint-selection protocol; small numerical differences are therefore interpreted cautiously.

The Cat-only setting served as a simple single-stream ROI reference, whereas ConcatFusion served as a non-adaptive multi-part ROI temporal baseline. Together, these comparisons clarified the contributions of detector robustness, complementary part modeling, missing-aware fusion, and temporal aggregation in PMTNet.

#### 3.2.1. Detector Variant Ablation

We first present the held-out test results of the three detector candidates retained from the detector-analysis stage, namely GIoU, WIoU v3, and CIoU + SimAM (Table 5). Although all three are competitive at the detection level, their downstream recognition performance is not equivalent. As shown in Table 5, CIoU + SimAM yields the strongest observed closed-loop result on the held-out test split, reaching 85.6% Top-1 Accuracy and 83.7% Macro-F1, compared with GIoU (81.8%/80.4%) and WIoU v3 (77.5%/74.9%).

This result further indicates that detector suitability for downstream behavior recognition cannot be judged by static detection quality alone. From the class-wise recalls, the advantage of CIoU + SimAM is mainly reflected in the more stable recognition of Rest, Grooming, and Active, while the recall for Locomotion remains high across all three candidates. The recall of Tail-Expressive is relatively unchanged under these detector variants, suggesting that this class remains intrinsically challenging even after detector improvement. Overall, CIoU + SimAM provides the strongest closed-loop behavior-recognition result among the compared detector variants and was therefore adopted in the finalized framework.

#### 3.2.2. Part-Wise Input Composition and Simple ROI Baseline

Table 6 reports the held-out test performance of four part-wise input compositions: Cat, Cat + head, Cat + tail, and Cat + head + tail. Here, the Cat-only setting serves as a simple single-stream ROI baseline. The results show that using only the global cat stream is insufficient, whereas adding part-level streams yields substantial gains.

A closer inspection reveals that different parts contribute differently. Relative to the Cat-only baseline (71.9%/63.7%), adding the head stream substantially improves overall recognition, particularly for Rest, Locomotion, and Active, indicating that head motion and local appearance cues are informative for broader behavioral context. In contrast, adding the tail stream alone yields a more targeted benefit for Tail-Expressive, where recall rises from 11.8% in the Cat-only setting to 47.1% in the Cat + tail setting. However, Cat + tail alone is not sufficient to match the global performance of Cat + head. The highest performance among the compared variants is achieved when all three streams are used jointly, indicating that head and tail cues are complementary rather than redundant. This result suggests that the gain of the framework comes from explicit anatomical decomposition rather than from cropping the cat region alone.

#### 3.2.3. Temporal Sequence Encoder Ablation

Table 7 compares the two temporal encoders introduced in Section 2.5, namely TCN and GRU, under the same detector outputs, part composition, fusion strategy, and classifier setting. TCN yields the stronger overall result, reaching 85.6% Top-1 Accuracy and 83.7% Macro-F1, compared with 83.1% and 80.8% for GRU.

The class-wise results indicate that the two encoders have different biases. The GRU yields slightly higher recall for Grooming and Tail-Expressive, suggesting that recurrent accumulation can be helpful for some temporally asymmetric or locally expressive behaviors. However, this advantage is offset by a notable drop in Locomotion recall, which decreases from 94.9% with TCN to 76.9% with GRU. By contrast, TCN provides a more balanced recognition profile across categories and is therefore adopted in the finalized framework.

#### 3.2.4. Fusion Ablation and Strong ROI Temporal Baseline

To isolate the effect of adaptive fusion, we compared the strong non-adaptive ROI temporal baseline, ConcatFusion, with the proposed GateFusion under the same detector outputs, three-part inputs, and missing-aware masking protocol. At the overall test-set level, GateFusion improves the overall result from 85.6% to 88.1% in Top-1 Accuracy and from 83.7% to 86.6% in Macro-F1 under the final evaluation setting.

Class-wise recalls show more clearly which categories benefit most from GateFusion. GateFusion improves Locomotion recall from 94.9% to 100.0%, Active from 87.5% to 91.7%, and Tail-Expressive from 58.8% to 64.7%, while maintaining strong performance on the remaining classes. Together with the part-wise results in Table 6, this comparison suggests that the improvement is not only associated with three-part ROI extraction, but also with the use of adaptive fusion over temporally unstable part streams.

To further examine whether missing-aware fusion was more beneficial under unstable part visibility, we grouped the 160 held-out test videos according to the missingness ratio of each part and compared GateFusion with ConcatFusion within each group. The missingness ratio was defined as the proportion of sampled frames in which the corresponding part was invalid or missing. The results are summarized in Table 8.

Across the test videos, the average missingness ratio was 0.33% for the cat body, 6.61% for the head, and 18.55% for the tail, confirming that tail visibility is substantially less stable than body and head visibility. The cat body was missing in very few videos, with 156 of 160 videos falling into the low-missingness group and no video falling into the high-missingness group. Therefore, cat missingness was not used as the main basis for interpreting fusion robustness.

The most informative pattern was observed for tail missingness. When the tail was almost always visible, ConcatFusion performed better than GateFusion by 4.42 percentage points. However, as tail missingness increased, GateFusion became more advantageous: it outperformed ConcatFusion by 2.96 percentage points in the medium tail-missingness group and by 8.28 percentage points in the high tail-missingness group. This indicates that GateFusion is not necessarily superior when all part evidence is complete, but its advantage becomes clearer when tail evidence is frequently missing or unreliable. A correlation analysis showed the same tendency. Tail missingness had a weaker negative association with GateFusion accuracy than with ConcatFusion accuracy, with Pearson and Spearman correlations of −0.052 and −0.102 for GateFusion, compared with −0.157 and −0.302 for ConcatFusion.

Head missingness showed a less consistent pattern. GateFusion performed better in the high head-missingness group, but this group contained only 11 videos, while ConcatFusion performed better in the medium head-missingness group. The overall test-set comparison between ConcatFusion and GateFusion is summarized in Table 9.

#### 3.2.5. Temporal Readout and Sequence Aggregation Ablation

Finally, we compared three clip-level aggregation strategies: last-step aggregation, mean aggregation, and attention aggregation under the final evaluation protocol. As summarized in Table 10, the choice of temporal readout had a substantial impact on final recognition quality. To assess run-to-run stability, we further repeated each temporal readout setting with three independent random seeds, and the corresponding test results are summarized in Table 11 as mean ± standard deviation; for each seed, the final checkpoint was selected by the highest validation Macro-F1. This readout comparison was selected for explicit variability reporting because it directly determines the final PMTNet configuration and includes relatively close-performing alternatives. Table 10 reports the detailed class-wise results of the best observed run, whereas Table 11 summarizes run-to-run variability for the same readout choices. The single-run results in Table 10 were used to show the detailed class-wise behavior of the best observed configuration, whereas Table 11 was used to characterize the stability of the readout comparison across seeds. Among the three alternatives, attention aggregation achieved the highest observed single-run performance, with 93.1% Top-1 Accuracy and 90.9% Macro-F1, and also showed the strongest mean performance across three independent runs in Table 11. Mean aggregation provided a strong reference, whereas last-step aggregation remained consistently inferior. Because formal significance testing was not performed, the emphasis here was on the consistent ranking and performance trend rather than on claiming statistical significance for every pairwise difference.

Last-step aggregation performed clearly worse than mean and attention aggregation, while attention aggregation achieved the highest observed performance among the three readout strategies. This result shows that the choice of temporal readout has a substantial influence on the final recognition performance.

**Figure 6:**
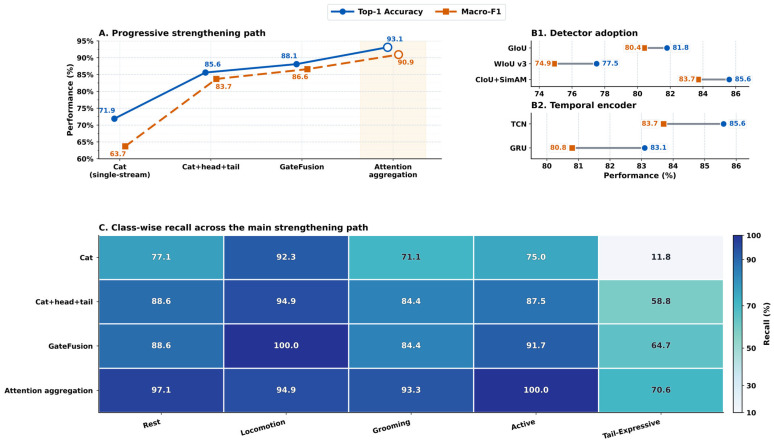
Summary of Stage-3 component comparisons. Panels (**A**,**B1**,**B2**) compare Top-1 Accuracy and Macro-F1 across component choices, while Panel (**C**) summarizes class-wise recall across representative settings. Darker cells in Panel (**C**) indicate higher recall.

Figure 6 provides a compact summary of the Stage-3 component comparisons. Across these comparisons, progressively stronger performance is obtained by moving from the Cat-only reference to complementary three-part inputs, from non-adaptive ConcatFusion to GateFusion, and from simpler temporal readout to attention-based aggregation. These results suggest that the final PMTNet is likely supported by the combined contribution of robust part detection, complementary part modeling, missing-aware fusion, and temporal aggregation.

**Figure 7:**
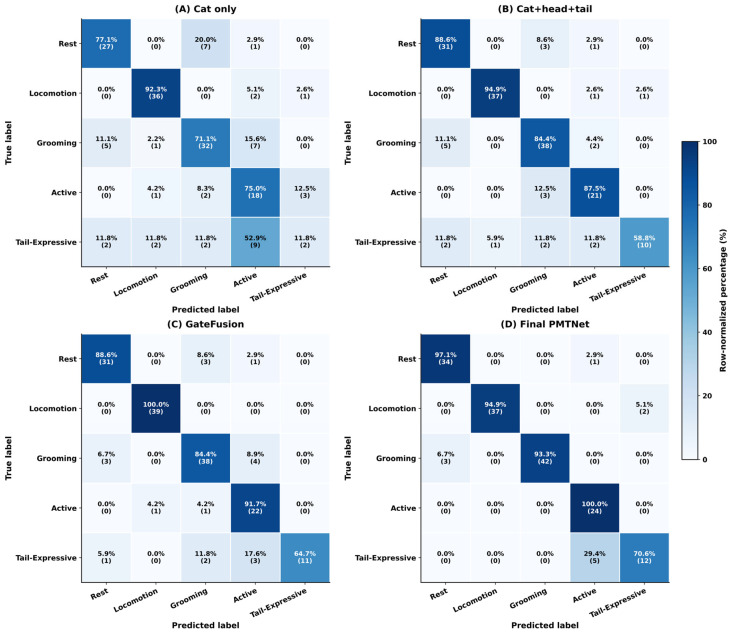
Confusion-matrix heatmaps of representative Stage-3 models: (**A**) Cat only, (**B**) Cat + head + tail, (**C**) GateFusion, and (**D**) final PMTNet. Rows denote ground-truth classes and columns denote predicted classes; diagonal cells indicate correct predictions, and off-diagonal cells indicate confusion.

Figure 7 visualizes the class-wise error patterns of representative Stage-3 configurations, including the Cat-only baseline, the three-part Cat + head + tail setting, the GateFusion model, and the final PMTNet with attention-based temporal aggregation. Consistent with Table 5, Table 6, Table 7 and Table 8, the confusion matrices show reduced cross-class confusion, especially for Grooming, Active, and Tail-Expressive, while Locomotion remains relatively stable across configurations. The final PMTNet therefore uses CIoU + SimAM for detection, cat/head/tail as part inputs, GateFusion for fusion, TCN for temporal modeling, and attention aggregation for clip-level readout. The confusion matrices show that Locomotion was well separated in most settings, while remaining errors mainly involved locally expressed behaviors such as Grooming and Tail-Expressive. The final PMTNet reduced these errors but did not fully remove them.

The confusion matrices show that Locomotion is relatively well separated across most settings, whereas residual errors are more often associated with locally expressed behaviors such as Grooming and Tail-Expressive. In the final PMTNet setting, these confusions are reduced but not fully eliminated.

#### 3.2.6. Qualitative Analysis of Successful and Failed Cases

**Figure 8:**
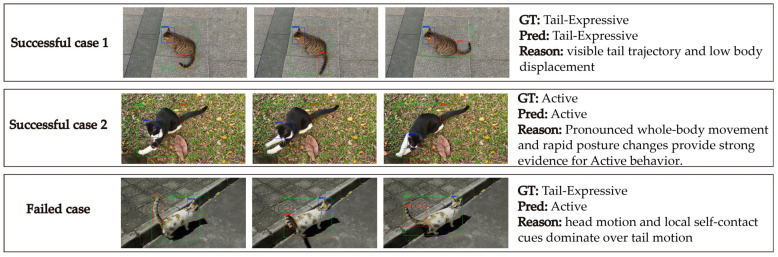
Each row shows three sampled frames from one test video clip, with detected cat, head, and tail regions overlaid on the frames. The first row shows a successful Tail-Expressive case, where the tail remains visible and shows clear lateral movement while body displacement is limited. The second row shows a successful Active case, where pronounced whole-body movement and rapid posture changes support the final prediction. The third row shows a failed case in which a Tail-Expressive clip is misclassified as Active because head motion and local self-contact cues dominate over the tail-motion evidence.

To provide a more intuitive interpretation of the image/video-based behavior analysis results, we further visualize representative successful and failed detection-recognition cases in Figure 8. Each example shows three sampled frames from the same video clip, together with the detected cat, head, and tail regions and the final clip-level prediction. These examples are selected from the held-out test split and are used only for qualitative interpretation after model evaluation.

In the successful Tail-Expressive case, the tail remains visible across sampled frames and shows a clear lateral movement pattern while the cat body remains largely stationary. This provides consistent tail-specific evidence for the final prediction. In the successful Active case, pronounced whole-body movement and rapid posture changes provide stronger evidence for Active behavior, showing that PMTNet can also rely on global body dynamics when the behavior is not primarily tail-driven.

The failed case illustrates a typical source of confusion for Tail-Expressive clips. Although tail motion is present, head motion and local self-contact cues become more visually dominant in the sampled sequence, causing the clip to be misclassified as Active. This example is consistent with the remaining class-wise errors shown in Figure 7. The relatively lower recall for Tail-Expressive reflects the difficulty of recognizing subtle tail-dominant behaviors when tail visibility is intermittent, the tail occupies only a small image region, or multiple behavioral cues co-occur within the same clip. These errors also indicate a current limitation of PMTNet: its recognition of tail-dominant behavior still depends on the quality and temporal continuity of tail localization and on the balance between tail cues and competing head/body motion cues.

### 3.3. Comparison with Representative End-to-End Video Recognition Baselines

The baseline comparison protocol and implementation settings are described in Section 2.6. In this section, we report the held-out test performance of PMTNet and the representative baselines.

As shown in Table 12, PMTNet achieves the best overall performance, reaching 93.1% Top-1 Accuracy and 90.9% Macro-F1. Among the full-frame global baselines, TimeSformer with AdamW provides the strongest comparator at 84.4%/85.8%, followed by VideoMAE at 74.3%/70.4% and SlowFast at 72.5%/70.6%. TimeSformer with SGD is reported mainly to show optimization sensitivity on the present dataset. Importantly, the advantage of PMTNet is not established only against full-frame baselines: within the same ROI-based setting, both the Cat-only baseline (71.9%/63.7%) and the stronger ConcatFusion baseline (85.6%/83.7%) remain below the final PMTNet configuration.

The class-wise results show that the compared global video baselines exhibit different category-dependent patterns. SlowFast performs strongly on Rest (100.0%) and reasonably well on Locomotion (84.6%), but obtains lower recall on Grooming (51.1%) and Tail-Expressive (41.2%). VideoMAE reaches 100.0% recall on Active and remains competitive on Rest (91.4%) and Locomotion (87.1%), but its Tail-Expressive recall is lower (29.4%). TimeSformer with AdamW provides the strongest global baseline, with high recall on Locomotion (100.0%) and Tail-Expressive (94.1%), although its Grooming recall remains lower than that of PMTNet (60.0% vs. 93.3%). Overall, under the present evaluation setting, PMTNet shows the highest overall performance and a relatively balanced class-wise profile among the compared methods.

These comparisons provide the empirical basis for the broader interpretation discussed in Section 4. Although the full detector-driven pipeline contains 31.64 M parameters in total, the upstream DEIM detector is frozen and executed offline for part extraction; consequently, online inference mainly relies on the lightweight downstream temporal recognizer, which has only 0.929 M parameters. For completeness, the model complexity and runtime efficiency of the representative baselines and PMTNet under the current evaluation setting are summarized in Table 13. It should be noted that the PMTNet efficiency reported here corresponds only to the lightweight Stage-3 recognizer under the offline cached-feature setting, and is therefore not directly comparable to the end-to-end raw-video baselines.

From an application-oriented perspective, the class-wise recalls in Table 12 also indicate that different misclassifications may have different practical consequences. Errors involving Grooming or Tail-Expressive clips may be more relevant to welfare-oriented monitoring, whereas confusion between Locomotion and Active mainly affects the interpretation of activity level. Therefore, the reported performance should be interpreted as benchmark-level evidence for the feasibility of part-centric video analysis, rather than as sufficient validation for fully autonomous real-world decision-making.

## 4. Discussion

The present study was motivated by a central challenge in cat behavior recognition: in unconstrained videos, behavior semantics are often carried by highly deformable and intermittently visible anatomical parts, whereas conventional full-frame video models tend to emphasize global appearance and background context. The results are consistent with our hypothesis that robust cat behavior recognition can benefit from stable part-centric temporal evidence rather than from stronger global spatiotemporal modeling alone. This finding agrees with previous animal-video studies showing that recognition in diverse real-world animal videos remains challenging for general full-frame action models, and with reports that video models may rely on static scene or background context when action-relevant cues are subtle. Our results extend these observations to feline behavior recognition by showing that body, head, and tail streams can provide useful local evidence when behavior semantics are carried by small, deformable, or intermittently visible anatomical parts. Thus, under the present dataset and evaluation protocol, the observed advantage of PMTNet may be related to the match between its part-centric design and the anatomical-temporal characteristics of cat behavior.

From a practical perspective, PMTNet may be most useful as a non-invasive screening or decision-support tool rather than as a fully autonomous diagnostic system. Because the framework relies on ordinary video input and does not require wearable sensors, it has potential value for welfare monitoring, veterinary observation, and long-term behavior screening in homes, clinics, or shelters. However, welfare-relevant predictions should still be reviewed by humans, especially when misclassification may hide changes in grooming behavior or tail-dominant expression. Therefore, the present results should be interpreted as benchmark-level evidence of feasibility rather than proof of immediate clinical or commercial deployment.

A key finding is that detector quality for downstream behavior recognition cannot be judged by static image-domain metrics alone. This finding is aligned with prior work highlighting the difficulty of stable animal pose or part localization under occlusion, deformation, and cross-species variation. In our detector comparison, WIoU v3 achieved stronger AP/AP75-oriented performance, yet CIoU + SimAM delivered the best closed-loop recognition result in Stage 3. This discrepancy suggests that, for behavior recognition, the temporal usability of detections may be at least as important as isolated frame-level box quality. A detector may obtain strong overlap on easier retained frames while still dropping small parts such as the tail under rapid articulation or motion blur. Such intermittent missingness may be especially harmful for sequence modeling, because downstream recognition relies on continuous part evidence. These results support the use of a downstream-aware evaluation protocol, in which detector selection considers continuity, missingness, and stability in the target video domain rather than mAP alone.

The part-wise and fusion ablations further clarify the role of local anatomical evidence in the proposed framework. This result is broadly consistent with part-based and region-based visual analysis, where object or body-part regions can reduce background interference and preserve task-relevant local cues. In our experiments, the lower performance of the global cat stream and the improvement obtained by adding head and tail streams indicate that local anatomical cues provide complementary information rather than redundant features. In the present five-class dominant-behavior taxonomy, the cat body stream captures overall posture and body displacement, the head stream provides cues related to orientation changes and self-directed motion, and the tail stream supplies the defining evidence for Tail-Expressive behavior. The improvement from ConcatFusion to GateFusion further suggests that simply extracting parts is not sufficient for unconstrained feline videos; missing and unreliable part observations need to be handled during temporal fusion. Therefore, the cat/head/tail design is a task-oriented representation intended to capture the principal behavioral cues required by the current classification setting.

Detection errors can propagate to behavior recognition through missing ROI features and noisy geometric motion descriptors. In PMTNet, this propagation is explicitly represented by part-level visibility masks. When a cat, head, or tail detection falls below the confidence threshold or has invalid geometry, the corresponding part is marked as missing, its ROI contribution is masked, and its geometric motion descriptor is zeroed. When a detection is retained but spatially unstable, the extracted ROI appearance feature and relative geometric motion cue may still become noisy. Therefore, missingness and bounding-box instability are the two main measurable forms of upstream detector-error propagation.

The detector-variant ablation provides a closed-loop view of this effect: under the same Stage-3 recognizer, different upstream detectors lead to different behavior-recognition performance, indicating that final recognition is sensitive to detector quality. The missingness-stratified analysis further shows that GateFusion is more beneficial in videos with medium or high tail-missing rates, suggesting that the missing-aware design reduces the impact of unreliable tail observations. Nevertheless, this mechanism does not fully eliminate upstream detection errors. The lack of an independent image-level detection test set is therefore a limitation of the present study, and future work should include a separate detection test set or external detection benchmark to more fully quantify detector generalization and error propagation.

Finer anatomical regions, such as paws, legs, ears, and the mouth/nose area, may provide additional information in ambiguous cases or in more fine-grained feline behavior analysis. Some of these cues are already partially included within the existing ROIs, with paw and leg information contained in the cat body region and mouth, nose, and ear cues partly covered by the head region. They are not modeled as independent streams in the present study because the current five-class taxonomy does not require these subregions as defining class cues, and because reliable localization of very small parts would increase annotation cost and detection instability in unconstrained videos. Future extensions of PMTNet could incorporate additional part streams or lightweight keypoint/pose cues when the task is expanded toward finer-grained behavior categories.

The temporal ablations provide additional insight into how cat behavior can be modeled over time. The stronger performance of TCN is consistent with the general role of temporal convolutions in efficiently capturing local motion patterns, whereas GRU provides a recurrent alternative for accumulating sequential evidence. In the present task, the comparison extends this general distinction to detector-derived part streams, suggesting that stable local temporal pattern extraction may be particularly useful for this setting, although this conclusion should be interpreted within the current dataset and model scope. The weaker performance of last-step aggregation also suggests that behavior-relevant evidence is not always concentrated near the end of a clip. The improvement from attention aggregation may reflect the uneven temporal distribution of discriminative cues, allowing the model to emphasize informative sub-segments rather than treating all time steps uniformly.

The comparison with end-to-end video backbones further positions PMTNet relative to common full-frame video recognition models. Although global video models have shown strong performance in human-centered action benchmarks [48], previous animal-video studies have reported that real-world animal behavior recognition remains challenging under diverse viewpoints, backgrounds, and motion patterns. Our results are consistent with this broader observation and further suggest that the challenge may be more pronounced when class semantics depend on localized anatomical motion rather than whole-frame activity. In this sense, PMTNet differs from full-frame baselines by explicitly preserving body, head, and tail evidence that may otherwise be diluted by background or whole-frame context. Taken together, the baseline comparisons suggest that the observed advantage of PMTNet may be related to a better match between the model design and the part-dependent nature of cat behavior under the present dataset and evaluation protocol.

Several limitations should nevertheless be noted. First, although the dataset includes heterogeneous real-world videos, all experiments were conducted on a single task-specific benchmark. Because PMTNet was trained and evaluated on standardized short clips, its reported performance should be interpreted as short-clip dominant-behavior recognition rather than continuous long-video behavior analysis. Therefore, the current results mainly demonstrate within-dataset performance and should not be interpreted as evidence of broad generalization across different environments, cat populations, camera settings, or species. The dataset also remains moderate in scale and is intentionally limited to a compact five-class scope, which defines a clear study boundary rather than an exhaustive behavioral taxonomy. Tail-Expressive remains the smallest and most challenging class. Even with explicit tail modeling, this category is affected by residual detector instability and by the intrinsic difficulty of the cue itself: tail motion is often small, easily blurred or partially occluded, and may appear only briefly. The boundary between Tail-Expressive and low-displacement Rest or Locomotion clips can also be ambiguous in short real-world videos. Although the annotation protocol uses explicit class definitions and deterministic precedence rules, and showed high inter-annotator agreement, clip-level single-label annotation may still involve some subjectivity, especially for mixed or transitional behaviors. Second, the current pipeline is decoupled and relies on offline cached detector outputs. This improves efficiency and experimental control, but detector errors are propagated forward rather than corrected jointly with the recognizer. Although the architecture is compatible with online sequential inference, the present study evaluates it mainly in an offline cached-feature setting rather than as a fully deployed real-time system. Third, because the self-collected footage was obtained through opportunistic real-world recording, no exhaustive registry of individual cat identities was maintained across the full corpus. Leakage control therefore relies primarily on conservative isolation by source, raw video, recording session, and stable scene context rather than on global identity verification of every cat. We consider this design appropriate for the present task-oriented benchmark, but no independent external dataset was used in this study. Broader external validation is therefore necessary before drawing stronger conclusions about cross-environment, cross-population, or cross-species generalization. Statistical robustness should also be considered when interpreting the results. Although several Stage-3 comparisons were repeated during model development and showed largely consistent trends, systematic variability measures are reported in the main text only for the temporal readout experiment. Other key comparisons, including detector variants, temporal encoders, fusion strategies, and global video baselines, are presented mainly as representative results under a fixed split. Therefore, small performance gaps should not be over-interpreted as statistically definitive. Finally, the architectural scope of the Stage-3 recognizer is limited to lightweight TCN and GRU encoders; transformer-based temporal encoders over part-level ROI streams are left for future investigation.

Future work can address these limitations in several directions. Larger and more diverse datasets would help evaluate generalization across breeds, viewpoints, environments, and more fine-grained or composite behaviors. The task could also be extended from clip-level single-label recognition to multi-label recognition or temporal localization, which would better reflect the continuity and overlap of real animal behavior. On the modeling side, uncertainty-aware fusion, stronger temporal refinement for small-part trajectories, and end-to-end joint optimization between detection and recognition may further improve robustness under severe occlusion and motion blur. Although this study focuses on cats, the proposed design principles—downstream-aware detector selection, missing-aware part fusion, and selective temporal aggregation—may also generalize to other animal behavior-recognition problems involving articulated bodies and unstable local visibility.

## 5. Conclusions

This study presented PMTNet, a part-centric temporal framework for cat behavior recognition in unconstrained real-world videos. Under the current benchmark and evaluation setting, the results suggest that explicitly modeling body, head, and tail evidence can improve recognition when behavior cues are localized, deformable, and intermittently visible. PMTNet achieved 93.1% Top-1 Accuracy and 90.9% Macro-F1, showing stronger and more balanced performance than the compared video recognition baselines on the present dataset. From an application perspective, this framework may support non-invasive cat behavior analysis in scenarios such as animal welfare monitoring, veterinary observation, and long-term behavioral screening in domestic, clinical, or shelter environments. Nevertheless, these findings should be interpreted within the current dataset scope, and further external validation is needed before broader deployment. Future work should examine larger and more diverse datasets, finer-grained or multi-label behavior settings, and tighter integration between detection and temporal recognition.

## Figures and Tables

**Table 1:** Task-oriented five-class taxonomy used for clip-level dominant-behavior annotation in cat videos.

ID	Class	Short Definition
0	Rest	Mostly stationary with minimal motion; no sustained locomotion, grooming, or active movement.
1	Locomotion	Sustained body translation (e.g., walking/running), regardless of camera motion.
2	Grooming	Self-cleaning actions dominate (e.g., licking fur/paws, scratching).
3	Active	High-energy movements dominate (e.g., pouncing, jumping, chasing, rapid turns).
4	Tail-Expressive	Tail motion is the dominant cue while overall body displacement is low (e.g., repeated tail swishing/flicking).

**Table 2:** Class-wise distribution of the cat behavior video dataset across training, validation, and test splits.

Split	Rest	Locomotion	Grooming	Active	Tail-Expressive	Total
Train	249	174	315	151	80	969
Validation	42	28	41	27	16	154
Test	35	39	45	24	17	160
Total	326	241	401	202	113	1283

**Table 3:** Main training settings of full-frame video baselines for reproducibility.

Model	Initialization	Optimizer	Learning Rate	Weight Decay	Epochs	Batch Size	Input
SlowFast	Kinetics-400	SGD	0.1	1 × 10^−4^	30	8	Official SlowFast recipe
TimeSformer (SGD)	Kinetics-400	SGD	0.005	1 × 10^−4^	15	4	8 frames, 224
TimeSformer (AdamW)	Kinetics-400	AdamW	1 × 10^−4^	0.05	15	4	8 frames, 224
VideoMAE	VideoMAE-base	AdamW	5 × 10^−5^	Framework default	50	16	8 frames, 224

Parameters not explicitly overridden in the training script followed the default settings of the corresponding framework.

**Table 4:** Static image-domain detection performance and video-domain robustness of detector candidates on the cat video corpus.

Architecture	Method	AP	AP50	AP75	Miss (Cat)	Miss (Head)	Miss (Tail)	meanIoU (Cat)	rel meanIoU (Head)	rel meanIoU (Tail)
CNN	YOLOv8-m	76.10%	95.80%	82.72%	2.68%	13.55%	37.25%	96.13%	88.27%	87.93%
DEIM	GIoU	76.36%	97.91%	85.18%	0.21%	7.62%	23.72%	93.47%	82.99%	79.18%
	DIoU	75.79%	97.34%	83.04%	0.17%	7.47%	24.93%	93.33%	82.40%	77.72%
	WIoU v3	77.22%	97.79%	85.41%	0.38%	7.39%	21.99%	93.33%	82.04%	77.57%
	CIoU	76.25%	98.26%	84.89%	0.17%	7.59%	22.94%	93.31%	81.87%	77.37%
	CIoU + CBAM	76.14%	97.56%	83.19%	0.16%	8.67%	24.00%	93.11%	81.79%	77.57%
	CIoU + SimAM	76.50%	97.76%	84.24%	0.04%	6.89%	23.25%	93.38%	81.99%	78.35%

**Table 5:** Closed-loop downstream behavior recognition under different detector variants.

Method	Top-1 Accuracy	Macro-F1	Rest (Recall)	Locomotion (Recall)	Grooming (Recall)	Active (Recall)	Tail-Expressive (Recall)
GIoU	81.8	80.4	77.1	94.9	77.8	91.7	58.8
WIoU v3	77.5	74.9	68.6	92.3	82.2	70.8	58.8
CIoU + SimAM	85.6	83.7	88.6	94.9	84.4	87.5	58.8

**Table 6:** Effect of part-wise input composition and simple ROI baseline comparisons on downstream cat behavior recognition.

Method	Top-1 Accuracy	Macro-F1	Rest (Recall)	Locomotion (Recall)	Grooming (Recall)	Active (Recall)	Tail-Expressive (Recall)
Cat	71.9	63.7	77.1	92.3	71.1	75.0	11.8
Cat + head	80.6	75.0	88.6	97.4	75.6	87.5	29.4
Cat + tail	73.1	70.4	77.1	76.9	75.6	75.0	47.1
Cat + head + tail	85.6	83.7	88.6	94.9	84.4	87.5	58.8

**Table 7:** Comparison of temporal sequence encoders in Stage-3 behavior recognition.

Method	Top-1 Accuracy	Macro-F1	Rest (Recall)	Locomotion (Recall)	Grooming (Recall)	Active (Recall)	Tail-Expressive (Recall)
TCN	85.6	83.7	88.6	94.9	84.4	87.5	58.8
GRU	83.1	80.8	85.7	76.9	88.9	87.5	70.6

**Table 8:** Effect of missing-aware fusion under different part-missingness levels on the held-out test split.

Part	Missingness Group	No. of Videos	Mean Missing Rate	GateFusion Mean Accuracy	ConcatFusion Mean Accuracy	Gate−Concat
cat	Low, 0 ≤ r ≤ 0.01	156	0.0	88.4	86.9	+1.54 pp
cat	Medium, 0.01 < r ≤ 0.4	4	13.3	25.0	70.0	−45.00 pp
cat	High, 0.4 < r ≤ 1	0	-	-	-	-
head	Low, 0 ≤ r ≤ 0.01	108	0.0	87.9	86.3	+1.67 pp
head	Medium, 0.01 < r ≤ 0.4	41	9.9	84.3	90.7	−6.34 pp
head	High, 0.4 < r ≤ 1	11	59.0	85.4	72.7	+12.73 pp
tail	Low, 0 ≤ r ≤ 0.01	77	0.0	91.9	96.3	−4.42 pp
tail	Medium, 0.01 < r ≤ 0.4	54	13.7	82.5	79.6	+2.96 pp
tail	High, 0.4 < r ≤ 1	29	76.7	81.3	73.1	+8.28 pp

The missingness ratio r is defined as the proportion of sampled frames in which the corresponding part was invalid or missing. Each video is evaluated over five independent runs, and the reported accuracy is the average per-video accuracy within each group. “pp” denotes percentage points.

**Table 9:** Comparison of fusion strategies for missing-aware multi-part behavior recognition.

Method	Top-1 Accuracy	Macro-F1	Rest (Recall)	Locomotion (Recall)	Grooming (Recall)	Active (Recall)	Tail-Expressive (Recall)
ConcatFusion	85.6	83.7	88.6	94.9	84.4	87.5	58.8
GateFusion	88.1	86.6	88.6	100.0	84.4	91.7	64.7

**Table 10:** Effect of temporal readout and sequence aggregation strategies on downstream behavior recognition.

Method	Top-1 Accuracy	Macro-F1	Rest (Recall)	Locomotion (Recall)	Grooming (Recall)	Active (Recall)	Tail-Expressive (Recall)
Last-step aggregation	73.1	70.2	80.0	84.6	71.1	66.7	47.1
Mean aggregation	88.1	86.6	88.6	100.0	84.4	91.7	64.7
Attention aggregation	93.1	90.9	97.1	94.9	93.3	100	70.6

**Table 11:** Robustness summary of temporal readout strategies over three independent runs.

Method	Top-1 Accuracy (Mean ± Std)	Test Macro-F1 (Mean ± Std)
Last-step aggregation	72.3 ± 5.7	68.0 ± 5.7
Mean aggregation	85.2 ± 2.9	82.6 ± 3.5
Attention aggregation	91.7 ± 2.0	89.1 ± 2.9

**Table 12:** Comparison between PMTNet and representative end-to-end video recognition baselines on the cat behavior dataset.

Method	Top-1 Accuracy	Macro-F1	Rest (Recall)	Locomotion (Recall)	Grooming (Recall)	Active (Recall)	Tail-Expressive (Recall)	Total Params
SlowFast	72.5	70.6	100	84.6	51.1	75	41.2	34.57 M
TimeSformer (SGD)	43.1	29.3	80	76.9	24.4	0	0	121.40 M
TimeSformer (AdamW)	84.4	85.8	97.1	100.0	60	79.2	94.1	121.40 M
VideoMAE	74.3	70.4	91.4	87.1	53.3	100	29.4	86.83 M
PMTNet	93.1	90.9	97.1	94.9	93.3	100	70.6	30.71 M (DEIM) + 0.93 M

Global baselines were initialized from official Kinetics-400 pre-trained checkpoints and fine-tuned on the same training/validation/test split. TimeSformer is reported with both its official SGD default setting and an AdamW variant to expose optimization sensitivity on the present dataset; the AdamW result is treated as the stronger primary comparator in the main discussion. PMTNet includes a frozen DEIM detector (30.71 M) and a lightweight Stage-3 recognizer (0.929 M).

**Table 13:** Model complexity and efficiency comparison on the current evaluation setting.

Method	Preprocess Overhead (ms)	Inference Latency (ms)	End-to-End Time (ms)	FPS (f/s)	GPU Memory (MB)
SlowFast	13812.93	218.5	14031.61	0.07	3138.16
TimeSformer(SGD)	3496.92	43.01	3540.06	0.29	697.69
TimeSformer (AdamW)	3517.89	43.57	3561.59	0.29	697.69
VideoMAE	841.34	17.66	860.04	1.17	406.97
PMTNet (Stage-3 only)	0.41	2.32	2.73	378.41	14.79

PMTNet efficiency is reported for the lightweight Stage-3 recognizer under the offline cached-feature setting, rather than the full detector-plus-recognizer pipeline; therefore, these numbers are not directly comparable to end-to-end raw-video baselines. End-to-End Time was measured independently and may not equal the sum of the preceding two columns.

## Data Availability

The original contributions presented in this study are included in the article. Further inquiries can be directed to the corresponding author.
